# Tetra­aqua­dodekakis-μ_2_-chlorido-di­iodido-*octa­hedro*-hexa­niobium(12 *Nb*—*Nb*) tetra­hydro­furan octa­solvate

**DOI:** 10.1107/S2414314622006186

**Published:** 2022-06-16

**Authors:** Florian Schröder, Martin Köckerling

**Affiliations:** a Universität Rostock, Institut für Chemie, Anorganische Festkörperchemie, Albert-Einstein-Str. 3a, D-18059 Rostock, Germany; Vienna University of Technology, Austria

**Keywords:** crystal structure, metal atom cluster, niobium, chloride, structure determination

## Abstract

The title compound, [Nb_6_Cl_12_I_2_(H_2_O)_4_]·8THF (THF is tetra­hydro­furan, C_4_H_8_O), comprises an uncharged niobium cluster unit surrounded by THF solvent mol­ecules. The edges of the {Nb_6_} octa­hedron are *μ*_2_-coordinated by twelve chlorido ligands. Four in-plane (equatorial plane) aqua ligands and two iodido ligands coordinating above and below the plane are bound at the corners of the {Nb_6_} atomic octa­hedron. O—H⋯O hydrogen bonds are formed between the aqua ligands and the THF solvent mol­ecules; one THF molecule is disordered over two positions with the major component having a site occupancy of 0.64 (2).

## Abstract 2

The isolated cluster unit in the structure of [Nb_6_Cl_12_I_2_(H_2_O)_4_]·8THF consists of an {Nb_6_} atom octa­hedron coordinated by twelve chlorido, four aqua, and two iodido ligands. THF solvent mol­ecules are bound *via* O—H⋯O hydrogen bonds.

## Graphical abstract

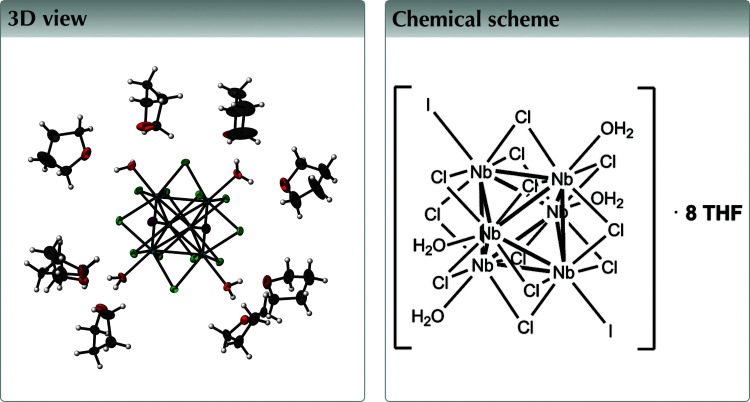

## Structure description

Cluster complexes of the early transition metals have been the subject of intense research for decades. Hexanuclear {Nb_6_} cluster complexes represent an inter­esting field of research (Cotton, 1964 ▸; Simon, 1988 ▸). Such compounds are produced *via* solid-state reactions at high temperatures and then converted into more soluble species by solvent chemistry (Koknat *et al.*, 1974 ▸; Lemoine *et al.*, 2019 ▸). The title compound can be obtained by dissolving [Nb_6_Cl_12_I_2_(H_2_O)_4_]·4H_2_O in THF and recrystallization.

**Figure 1:**
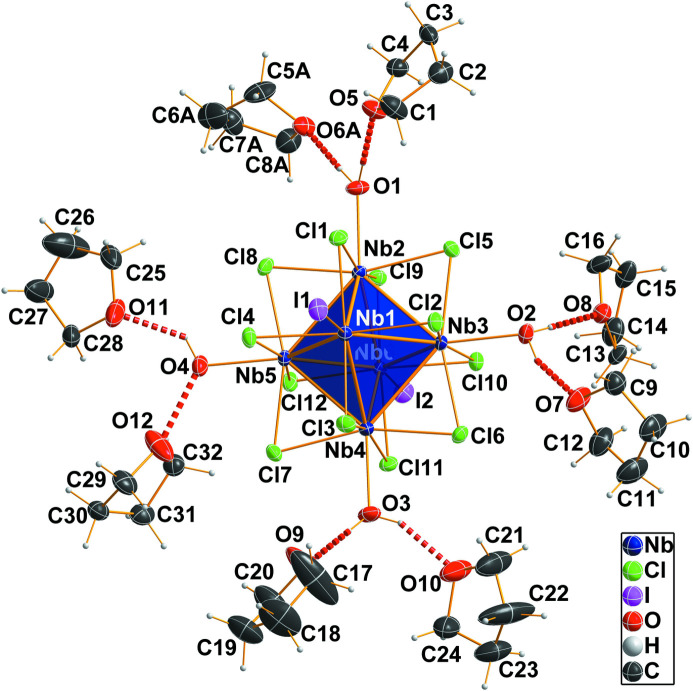
The discrete cluster unit of [Nb_6_Cl_12_I_2_(H_2_O)_4_]·8THF with surrounding THF solvent mol­ecules. Atoms are drawn as displacement ellipsoids at the 50% probability level. The {Nb_6_} metal atom octa­hedron is shown in a polyhedral representation, O—H⋯O hydrogen bonds are shown as red dashed lines. Of the disordered THF mol­ecule, only the major component (*A*) is shown for better visibility.

**Figure 2:**
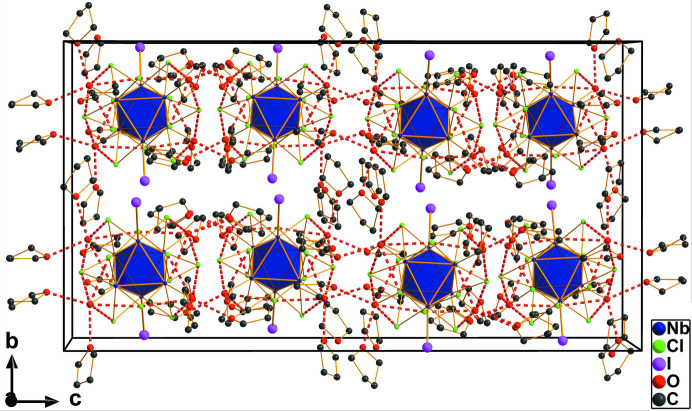
Arrangement of neutral cluster units and THF solvent mol­ecules in the unit cell in a view along the *a* axis. The {Nb_6_} metal atom octa­hedra are shown in a polyhedral representation, and O—H⋯O hydrogen bonds are shown as dashed red lines.

The {Nb_6_} atomic polyhedron is an octa­hedron (Fig. 1 ▸) in which two different Nb—Nb bond lengths have to be considered. The niobium atoms located in the equatorial plane (coordination by aqua ligands) have an average Nb_eq_—Nb_eq_ bond length of 2.896 Å. The niobium atoms above and below this plane (Nb_ax_), which are coordinated by iodido ligands, have Nb_ax_—Nb_eq_ bond lengths averaging at 2.938 Å. Thereby, the {Nb_6_} atomic octa­hedron is elongated, reflected also by the atomic distances between opposite niobium atoms. Within the equatorial plane they are 4.095 Å on average, and 4.2150 (8) Å between the axial sites. The twelve chlorido ligands of the inner ligand sphere are *μ*_2_-bridging over the edges of the {Nb_6_} atom octa­hedron. The average Nb_eq_—Cl bond length is 2.469 Å and Nb_ax_—Cl is 2.460 Å. Of the six outer coordin­ation sites, four aqua ligands singly bond to the Nb_eq_ atoms and two iodido ligands to the Nb_ax_ atoms with average Nb—O and Nb—I bond lengths of 2.223 and 2.944 Å, respectively. These atom distances indicate a cluster unit with 16 cluster-based electrons. Thus, there is no change of the oxidation state compared to the starting material. Rather strong hydrogen bonds (Steiner, 2002 ▸) with donor⋯acceptor distances in the range 2.530 (8)–2.68 (5) Å are found between the aqua ligands of the {Nb_6_} unit and the O atoms of the solvent THF mol­ecules (Table 1 ▸). A view of the packing of cluster and THF solvent mol­ecules is given in Fig. 2 ▸.

## Synthesis and crystallization

Starting from the compound [Nb_6_Cl_12_I_2_(H_2_O)_4_]·4H_2_O (Schäfer *et al.*, 1972 ▸; Brničević *et al.*, 1981 ▸), the title compound [Nb_6_Cl_12_I_2_(H_2_O)_4_]·8THF can be synthesized in moderate yields. 50 mg (36.21 *μ*mol) of [Nb_6_Cl_12_I_2_(H_2_O)_4_]·4H_2_O and 3 ml (36.86 mmol) of THF were placed in a 4 ml vial and heated in a sand bath at 333 K for two days. From the dark-green solution, small black crystals formed together with a larger amount of an amorphous sediment. The crystals were washed several times with THF. 32 mg (16.97 *μ*mol, yield 64%) of [Nb_6_Cl_12_I_2_(H_2_O)_4_]·8THF were obtained.

## Refinement

Crystal data, data collection and structure refinement details are summarized in Table 2 ▸. One of the solvent THF mol­ecules, O6, C5–C8, is disordered over two sets of sites [ratio 0.64 (2):0.36 (2) for parts *A*:*B*], with constraints on some *U* ^ij^ parameters.

## Supplementary Material

Crystal structure: contains datablock(s) I. DOI: 10.1107/S2414314622006186/wm4165sup1.cif

Structure factors: contains datablock(s) I. DOI: 10.1107/S2414314622006186/wm4165Isup2.hkl

CCDC reference: 2178831

Additional supporting information:  crystallographic information; 3D view; checkCIF report

## Figures and Tables

**Table 1:** Hydrogen-bond geometry (Å, °)

*D*—H⋯*A*	*D*—H	H⋯*A*	*D*⋯*A*	*D*—H⋯*A*
O1—H1*A*⋯O5	0.85	1.79	2.642 (7)	180
O1—H1*B*⋯O6*A*_*a*	0.85	1.75	2.60 (3)	178
O1—H1*B*⋯O6*B*_*b*	0.85	1.83	2.68 (5)	173
O2—H2*A*⋯O7	0.85	1.83	2.639 (7)	158
O2—H2*B*⋯O8	0.85	1.78	2.634 (7)	180
O3—H3*B*⋯O9	0.85	1.75	2.601 (8)	179
O3—H3*A*⋯O10	0.85	1.88	2.637 (8)	148
O4—H4*B*⋯O11	0.85	1.92	2.613 (8)	138
O4—H4*A*⋯O12	0.85	2.09	2.530 (8)	112

**Table 2:** Experimental details

Crystal data	
Chemical formula	[Nb_6_Cl_12_I_2_(H_2_O)_4_]·8C_4_H_8_O
*M*_r_	1885.55
Crystal system, space group	Orthorhombic, *P* *b* *c* *a*
Temperature (K)	123
*a*, *b*, *c* (Å)	19.3389 (7), 18.1968 (7), 34.039 (1)
*V* (Å^3^)	11978.6 (8)
*Z*	8
Radiation type	Mo *K*α
μ (mm^−1^)	2.72
Crystal size (mm)	0.23 × 0.16 × 0.14

Data collection	
Diffractometer	Bruker APEXII CCD
Absorption correction	Multi-scan (*SADABS*; Krause *et al.*, 2015 ▸)
No. of measured, independent and observed [*I* > 2σ(*I*)] reflections	94479, 15885, 11673
*R*_int_	0.050
(sin θ/λ)_max_ (Å^−1^)	0.683

Refinement	
*R*[*F* ^2^ > 2σ(*F* ^2^)], *wR*(*F* ^2^), *S*	0.051, 0.132, 1.06
No. of reflections	15885
No. of parameters	602
H-atom treatment	H-atom parameters constrained
Δρ_max_, Δρ_min_ (e Å^−3^)	1.47, −1.50

Computer programs: *APEX3* and *SAINT* (Bruker, 2017 ▸), *SHELXS* (Sheldrick, 2008 ▸), *SHELXL* (Sheldrick 2015 ▸), *DIAMOND* (Brandenburg, 2019 ▸) and *publCIF* (Westrip, 2010 ▸).

## full crystallographic data

### Crystal data

**Table:**

[Nb_6_Cl_12_I_2_(H_2_O)_4_]·8C_4_H_8_O	*D*_x_ = 2.091 Mg m^−^^3^
*M**_r_* = 1885.55	Mo *K*α radiation, λ = 0.71073 Å
Orthorhombic, *P**b**c**a*	Cell parameters from 9812 reflections
*a* = 19.3389 (7) Å	θ = 2.5–29.0°
*b* = 18.1968 (7) Å	µ = 2.72 mm^−^^1^
*c* = 34.039 (1) Å	*T* = 123 K
*V* = 11978.6 (8) Å^3^	Block, black
*Z* = 8	0.23 × 0.16 × 0.14 mm
*F*(000) = 7328	

### Data collection

**Table:**

Bruker APEXII CCD diffractometer	11673 reflections with *I* > 2σ(*I*)
Radiation source: microfocus sealed tube	*R*_int_ = 0.050
φ and ω scans	θ_max_ = 29.0°, θ_min_ = 1.6°
Absorption correction: multi-scan (*SADABS*; Krause *et al.*, 2015)	*h* = −26→24
	*k* = −24→24
94479 measured reflections	*l* = −36→45
15885 independent reflections	

### Refinement

**Table:**

Refinement on *F*^2^	Primary atom site location: structure-invariant direct methods
Least-squares matrix: full	Secondary atom site location: difference Fourier map
*R*[*F*^2^ > 2σ(*F*^2^)] = 0.051	Hydrogen site location: mixed
*wR*(*F*^2^) = 0.132	H-atom parameters constrained
*S* = 1.06	*w* = 1/[σ^2^(*F*_o_^2^) + (0.0359*P*)^2^ + 173.2937*P*] where *P* = (*F*_o_^2^ + 2*F*_c_^2^)/3
15885 reflections	(Δ/σ)_max_ = 0.001
602 parameters	Δρ_max_ = 1.47 e Å^−^^3^
0 restraints	Δρ_min_ = −1.50 e Å^−^^3^

### Special details

**Table:**

Geometry. All esds (except the esd in the dihedral angle between two l.s. planes) are estimated using the full covariance matrix. The cell esds are taken into account individually in the estimation of esds in distances, angles and torsion angles; correlations between esds in cell parameters are only used when they are defined by crystal symmetry. An approximate (isotropic) treatment of cell esds is used for estimating esds involving l.s. planes.
Refinement. Hydrogen atoms were placed in idealized positions and refined using a riding model.

### Fractional atomic coordinates and isotropic or equivalent isotropic displacement parameters (Å^2^)

**Table:**

	*x*	*y*	*z*	*U*_iso_*/*U*_eq_	Occ. (<1)
Nb1	0.59644 (3)	0.17243 (3)	0.36689 (2)	0.0169 (1)	
Nb2	0.58974 (3)	0.31709 (3)	0.32845 (2)	0.0171 (1)	
Nb3	0.58856 (3)	0.30859 (3)	0.41313 (2)	0.0160 (1)	
Nb4	0.46988 (3)	0.21109 (3)	0.40986 (2)	0.0171 (1)	
Nb5	0.47127 (3)	0.21952 (3)	0.32502 (2)	0.0176 (1)	
Nb6	0.46288 (3)	0.35529 (3)	0.37133 (2)	0.0163 (1)	
Cl1	0.67822 (8)	0.22178 (9)	0.31834 (5)	0.0230 (3)	
Cl2	0.67647 (8)	0.21119 (9)	0.41928 (5)	0.0218 (3)	
Cl3	0.53495 (8)	0.09517 (8)	0.41480 (5)	0.0239 (3)	
Cl4	0.53829 (8)	0.10741 (9)	0.31292 (5)	0.0236 (3)	
Cl5	0.67003 (8)	0.38061 (9)	0.37308 (4)	0.0218 (3)	
Cl6	0.52879 (8)	0.25339 (9)	0.47005 (4)	0.0224 (3)	
Cl7	0.38863 (8)	0.14800 (9)	0.36529 (5)	0.0236 (3)	
Cl8	0.53035 (8)	0.2760 (1)	0.26799 (4)	0.0244 (3)	
Cl9	0.52292 (8)	0.43133 (9)	0.32239 (4)	0.0220 (3)	
Cl10	0.52290 (8)	0.42292 (8)	0.42386 (4)	0.0204 (3)	
Cl11	0.38214 (8)	0.30702 (9)	0.42091 (4)	0.0216 (3)	
Cl12	0.38156 (8)	0.31507 (9)	0.32027 (4)	0.0229 (3)	
I1	0.68739 (2)	0.04382 (3)	0.36266 (2)	0.0287 (1)	
I2	0.36838 (2)	0.48292 (3)	0.37360 (2)	0.0274 (1)	
O1	0.6562 (2)	0.3756 (3)	0.2855 (1)	0.028 (1)	
H1A	0.6990	0.3682	0.2815	0.042*	
H1B	0.6507	0.3929	0.2625	0.042*	
O2	0.6540 (2)	0.3566 (3)	0.4607 (1)	0.024 (1)	
H2A	0.6462	0.3344	0.4822	0.035*	
H2B	0.6566	0.4015	0.4674	0.035*	
O3	0.4028 (3)	0.1542 (3)	0.4537 (2)	0.030 (1)	
H3A	0.4157	0.1653	0.4768	0.045*	
H3B	0.3908	0.1092	0.4535	0.045*	
O4	0.4085 (2)	0.1714 (3)	0.2773 (1)	0.029 (1)	
H4A	0.3749	0.1999	0.2721	0.044*	
H4B	0.4335	0.1661	0.2570	0.044*	
O5	0.7892 (3)	0.3526 (3)	0.2728 (2)	0.033 (1)	
C1	0.8400 (4)	0.3109 (5)	0.2931 (3)	0.045 (2)	
H1C	0.8578	0.2710	0.2761	0.054*	
H1D	0.8197	0.2887	0.3170	0.054*	
C2	0.8969 (4)	0.3623 (5)	0.3039 (2)	0.040 (2)	
H2C	0.9421	0.3367	0.3044	0.048*	
H2D	0.8885	0.3851	0.3299	0.048*	
C3	0.8943 (4)	0.4190 (4)	0.2714 (2)	0.034 (2)	
H3C	0.9113	0.4673	0.2807	0.041*	
H3D	0.9223	0.4034	0.2485	0.041*	
C4	0.8184 (4)	0.4224 (4)	0.2611 (2)	0.033 (2)	
H4C	0.7955	0.4632	0.2753	0.039*	
H4D	0.8122	0.4302	0.2325	0.039*	
O6A_a	0.643 (1)	0.430 (1)	0.2154 (8)	0.030 (4)	0.64 (2)
C5A_a	0.670 (3)	0.389 (3)	0.179 (2)	0.049 (6)	0.64 (2)
H5A_a	0.6924	0.3420	0.1866	0.059*	0.64 (2)
H5B_a	0.7026	0.4194	0.1639	0.059*	0.64 (2)
C6A_a	0.6005 (9)	0.3771 (8)	0.1577 (5)	0.060 (5)	0.64 (2)
H6A_a	0.6086	0.3633	0.1300	0.072*	0.64 (2)
H6B_a	0.5732	0.3379	0.1706	0.072*	0.64 (2)
C7A_a	0.565 (4)	0.447 (5)	0.160 (3)	0.044 (4)	0.64 (2)
H7A_a	0.5144	0.4417	0.1561	0.053*	0.64 (2)
H7B_a	0.5833	0.4831	0.1410	0.053*	0.64 (2)
C8A_a	0.5807 (4)	0.4710 (5)	0.2025 (3)	0.041 (2)	0.64 (2)
H8A_a	0.5895	0.5246	0.2036	0.050*	0.64 (2)
H8B_a	0.5411	0.4596	0.2199	0.050*	0.64 (2)
O6B_b	0.631 (3)	0.422 (3)	0.212 (2)	0.07 (2)	0.36 (2)
C5B_b	0.659 (5)	0.389 (6)	0.185 (3)	0.049 (6)	0.36 (2)
H5C_b	0.6436	0.3372	0.1851	0.059*	0.36 (2)
H5D_b	0.7098	0.3901	0.1886	0.059*	0.36 (2)
C6B_b	0.639 (1)	0.427 (1)	0.1452 (6)	0.038 (7)	0.36 (2)
H6C_b	0.6681	0.4699	0.1385	0.045*	0.36 (2)
H6D_b	0.6362	0.3925	0.1228	0.045*	0.36 (2)
C7B_b	0.566 (7)	0.449 (8)	0.162 (5)	0.044 (4)	0.36 (2)
H7C_b	0.5457	0.4902	0.1470	0.053*	0.36 (2)
H7D_b	0.5338	0.4068	0.1614	0.053*	0.36 (2)
C8B_b	0.5807 (4)	0.4710 (5)	0.2025 (3)	0.041 (2)	0.36 (2)
H8C_b	0.5976	0.5223	0.2039	0.050*	0.36 (2)
H8D_b	0.5396	0.4655	0.2196	0.050*	0.36 (2)
O7	0.6621 (3)	0.2999 (4)	0.5319 (2)	0.043 (1)	
C9	0.7253 (4)	0.2924 (5)	0.5529 (2)	0.038 (2)	
H9A	0.7568	0.3337	0.5468	0.046*	
H9B	0.7485	0.2458	0.5459	0.046*	
C10	0.7064 (5)	0.2929 (7)	0.5958 (3)	0.056 (3)	
H10A	0.7235	0.2479	0.6090	0.068*	
H10B	0.7262	0.3364	0.6091	0.068*	
C11	0.6288 (5)	0.2956 (7)	0.5964 (3)	0.061 (3)	
H11A	0.6089	0.2456	0.5986	0.073*	
H11B	0.6118	0.3260	0.6185	0.073*	
C12	0.6110 (5)	0.3297 (6)	0.5579 (3)	0.047 (2)	
H12A	0.5637	0.3158	0.5495	0.056*	
H12B	0.6142	0.3839	0.5593	0.056*	
O8	0.6622 (3)	0.4959 (3)	0.4812 (1)	0.029 (1)	
C13	0.6032 (4)	0.5277 (4)	0.5017 (2)	0.030 (2)	
H13A	0.6162	0.5420	0.5288	0.036*	
H13B	0.5646	0.4921	0.5030	0.036*	
C14	0.5828 (5)	0.5943 (5)	0.4780 (3)	0.043 (2)	
H14A	0.5618	0.6326	0.4949	0.052*	
H14B	0.5499	0.5812	0.4569	0.052*	
C15	0.6514 (5)	0.6195 (5)	0.4614 (2)	0.043 (2)	
H15A	0.6779	0.6484	0.4809	0.052*	
H15B	0.6449	0.6494	0.4374	0.052*	
C16	0.6869 (5)	0.5471 (5)	0.4522 (2)	0.041 (2)	
H16A	0.6747	0.5301	0.4255	0.050*	
H16B	0.7377	0.5523	0.4540	0.050*	
O9	0.3659 (4)	0.0167 (4)	0.4525 (3)	0.087 (3)	
C17	0.3983 (8)	−0.0391 (7)	0.4734 (6)	0.135 (9)	
H17A	0.4090	−0.0218	0.5003	0.161*	
H17B	0.4423	−0.0523	0.4603	0.161*	
C18	0.3548 (7)	−0.1018 (6)	0.4753 (5)	0.087 (4)	
H18A	0.3423	−0.1127	0.5029	0.104*	
H18B	0.3784	−0.1452	0.4640	0.104*	
C19	0.2917 (5)	−0.0831 (6)	0.4520 (4)	0.065 (3)	
H19A	0.2914	−0.1098	0.4266	0.078*	
H19B	0.2492	−0.0957	0.4667	0.078*	
C20	0.2968 (6)	−0.0021 (5)	0.4456 (4)	0.070 (4)	
H20A	0.2834	0.0106	0.4184	0.084*	
H20B	0.2660	0.0244	0.4640	0.084*	
O10	0.3910 (3)	0.1938 (5)	0.5279 (2)	0.064 (2)	
C21	0.4469 (5)	0.1819 (9)	0.5540 (3)	0.082 (4)	
H21A	0.4679	0.2295	0.5615	0.098*	
H21B	0.4828	0.1516	0.5410	0.098*	
C22	0.4213 (6)	0.145 (1)	0.5880 (4)	0.122 (8)	
H22A	0.4450	0.1624	0.6120	0.147*	
H22B	0.4275	0.0911	0.5857	0.147*	
C23	0.3459 (5)	0.1652 (7)	0.5885 (3)	0.065 (3)	
H23A	0.3182	0.1277	0.6026	0.078*	
H23B	0.3386	0.2136	0.6011	0.078*	
C24	0.3284 (5)	0.1673 (7)	0.5469 (3)	0.058 (3)	
H24A	0.3159	0.1178	0.5372	0.069*	
H24B	0.2892	0.2011	0.5421	0.069*	
O11	0.4333 (3)	0.1077 (5)	0.2100 (2)	0.063 (2)	
C25	0.4832 (4)	0.1328 (6)	0.1820 (3)	0.050 (2)	
H25A	0.5213	0.0968	0.1795	0.060*	
H25B	0.5028	0.1805	0.1903	0.060*	
C26	0.4469 (7)	0.141 (1)	0.1446 (4)	0.106 (6)	
H26A	0.4438	0.1932	0.1374	0.127*	
H26B	0.4729	0.1147	0.1237	0.127*	
C27	0.3818 (6)	0.1113 (9)	0.1478 (3)	0.079 (4)	
H27A	0.3780	0.0664	0.1315	0.095*	
H27B	0.3469	0.1472	0.1385	0.095*	
C28	0.3694 (4)	0.0931 (5)	0.1902 (2)	0.039 (2)	
H28A	0.3319	0.1240	0.2010	0.047*	
H28B	0.3563	0.0408	0.1931	0.047*	
O12	0.2795 (3)	0.1498 (4)	0.2820 (3)	0.064 (2)	
C29	0.2461 (4)	0.0793 (5)	0.2839 (3)	0.043 (2)	
H29A	0.2488	0.0586	0.3108	0.052*	
H29B	0.2674	0.0442	0.2653	0.052*	
C30	0.1722 (4)	0.0958 (4)	0.2727 (2)	0.032 (2)	
H30A	0.1665	0.0983	0.2438	0.039*	
H30B	0.1400	0.0586	0.2835	0.039*	
C31	0.1614 (4)	0.1697 (5)	0.2915 (2)	0.037 (2)	
H31A	0.1467	0.1643	0.3192	0.044*	
H31B	0.1261	0.1984	0.2772	0.044*	
C32	0.2314 (4)	0.2065 (4)	0.2890 (2)	0.034 (2)	
H32A	0.2322	0.2427	0.2673	0.040*	
H32B	0.2423	0.2321	0.3139	0.040*	

### Atomic displacement parameters (Å^2^)

**Table:**

	*U*^11^	*U*^22^	*U*^33^	*U*^12^	*U*^13^	*U*^23^
Nb1	0.0160 (2)	0.0185 (3)	0.0163 (3)	−0.0005 (2)	−0.0003 (2)	−0.0025 (2)
Nb2	0.0162 (3)	0.0219 (3)	0.0134 (2)	−0.0015 (2)	0.0002 (2)	0.0005 (2)
Nb3	0.0174 (3)	0.0176 (3)	0.0131 (2)	−0.0013 (2)	−0.0016 (2)	−0.0010 (2)
Nb4	0.0176 (3)	0.0184 (3)	0.0152 (3)	−0.0019 (2)	0.0025 (2)	−0.0005 (2)
Nb5	0.0156 (3)	0.0223 (3)	0.0150 (3)	−0.0012 (2)	−0.0007 (2)	−0.0043 (2)
Nb6	0.0163 (2)	0.0190 (3)	0.0135 (2)	−0.0008 (2)	−0.0004 (2)	−0.0006 (2)
Cl1	0.0186 (7)	0.0289 (8)	0.0217 (7)	0.0012 (6)	0.0042 (6)	−0.0010 (6)
Cl2	0.0211 (7)	0.0231 (7)	0.0212 (7)	0.0010 (6)	−0.0057 (6)	−0.0016 (6)
Cl3	0.0259 (8)	0.0191 (7)	0.0267 (8)	−0.0008 (6)	0.0025 (6)	0.0020 (6)
Cl4	0.0210 (7)	0.0245 (8)	0.0254 (8)	0.0001 (6)	−0.0024 (6)	−0.0106 (6)
Cl5	0.0190 (7)	0.0250 (7)	0.0214 (7)	−0.0055 (6)	−0.0017 (6)	0.0001 (6)
Cl6	0.0286 (8)	0.0256 (8)	0.0131 (7)	−0.0015 (6)	0.0007 (6)	0.0003 (6)
Cl7	0.0197 (7)	0.0237 (7)	0.0273 (8)	−0.0061 (6)	0.0012 (6)	−0.0065 (6)
Cl8	0.0239 (7)	0.0360 (9)	0.0133 (7)	−0.0008 (7)	−0.0005 (6)	−0.0024 (6)
Cl9	0.0226 (7)	0.0237 (7)	0.0196 (7)	−0.0007 (6)	0.0006 (6)	0.0045 (6)
Cl10	0.0232 (7)	0.0190 (7)	0.0189 (7)	0.0001 (6)	−0.0029 (6)	−0.0029 (5)
Cl11	0.0213 (7)	0.0232 (7)	0.0203 (7)	0.0006 (6)	0.0063 (6)	−0.0009 (6)
Cl12	0.0188 (7)	0.0292 (8)	0.0206 (7)	0.0017 (6)	−0.0058 (5)	−0.0031 (6)
I1	0.0253 (2)	0.0272 (2)	0.0336 (2)	0.0051 (2)	−0.0037 (2)	−0.0057 (2)
I2	0.0281 (2)	0.0276 (2)	0.0266 (2)	0.0062 (2)	−0.0003 (2)	0.0003 (2)
O1	0.019 (2)	0.042 (3)	0.023 (2)	0.001 (2)	0.005 (2)	0.010 (2)
O2	0.031 (3)	0.023 (2)	0.017 (2)	−0.001 (2)	−0.004 (2)	−0.002 (2)
O3	0.035 (3)	0.028 (3)	0.026 (3)	−0.004 (2)	0.011 (2)	0.005 (2)
O4	0.021 (2)	0.042 (3)	0.025 (2)	−0.001 (2)	−0.003 (2)	−0.012 (2)
O5	0.021 (2)	0.034 (3)	0.044 (3)	−0.004 (2)	0.000 (2)	0.008 (2)
C1	0.034 (4)	0.043 (5)	0.058 (6)	−0.008 (4)	−0.011 (4)	0.015 (4)
C2	0.026 (4)	0.057 (5)	0.035 (4)	0.006 (4)	−0.003 (3)	0.004 (4)
C3	0.028 (4)	0.033 (4)	0.042 (4)	−0.008 (3)	0.004 (3)	−0.014 (3)
C4	0.027 (4)	0.039 (4)	0.032 (4)	−0.003 (3)	0.004 (3)	0.001 (3)
O6A_a	0.020 (6)	0.041 (7)	0.029 (8)	0.001 (5)	−0.006 (5)	0.010 (5)
C5A_a	0.04 (2)	0.065 (7)	0.05 (2)	0.02 (1)	0.021 (9)	0.00 (1)
C6A_a	0.08 (1)	0.033 (8)	0.07 (1)	−0.004 (8)	0.021 (9)	−0.005 (7)
C7A_a	0.041 (5)	0.047 (6)	0.045 (9)	−0.004 (5)	−0.015 (5)	0.001 (6)
C8A_a	0.036 (4)	0.049 (5)	0.039 (5)	0.011 (4)	−0.002 (4)	0.004 (4)
O6B_b	0.05 (3)	0.14 (4)	0.02 (2)	0.04 (2)	0.02 (2)	0.02 (2)
C5B_b	0.04 (2)	0.065 (7)	0.05 (2)	0.02 (1)	0.021 (9)	0.00 (1)
C6B_b	0.06 (2)	0.04 (1)	0.02 (1)	−0.01 (1)	0.007 (9)	−0.009 (8)
C7B_b	0.041 (5)	0.047 (6)	0.045 (9)	−0.004 (5)	−0.015 (5)	0.001 (6)
C8B_b	0.036 (4)	0.049 (5)	0.039 (5)	0.011 (4)	−0.002 (4)	0.004 (4)
O7	0.042 (3)	0.065 (4)	0.020 (3)	0.011 (3)	−0.005 (2)	0.007 (3)
C9	0.036 (4)	0.045 (5)	0.034 (4)	0.001 (4)	−0.005 (3)	−0.001 (4)
C10	0.043 (5)	0.095 (8)	0.030 (4)	0.009 (5)	−0.010 (4)	0.006 (5)
C11	0.056 (6)	0.094 (9)	0.033 (5)	0.011 (6)	0.002 (4)	0.013 (5)
C12	0.044 (5)	0.058 (6)	0.038 (5)	0.020 (4)	−0.001 (4)	−0.012 (4)
O8	0.031 (3)	0.029 (3)	0.026 (3)	0.000 (2)	0.002 (2)	−0.005 (2)
C13	0.034 (4)	0.037 (4)	0.020 (3)	0.002 (3)	0.006 (3)	−0.008 (3)
C14	0.055 (5)	0.037 (4)	0.039 (5)	0.010 (4)	−0.004 (4)	−0.008 (4)
C15	0.073 (6)	0.030 (4)	0.027 (4)	−0.001 (4)	−0.001 (4)	−0.002 (3)
C16	0.054 (5)	0.037 (4)	0.032 (4)	−0.005 (4)	0.021 (4)	−0.001 (3)
O9	0.065 (5)	0.043 (4)	0.154 (9)	−0.030 (4)	−0.041 (5)	0.040 (5)
C17	0.09 (1)	0.056 (8)	0.25 (2)	−0.022 (7)	−0.11 (1)	0.07 (1)
C18	0.082 (9)	0.045 (6)	0.13 (1)	0.005 (6)	−0.017 (8)	0.040 (7)
C19	0.044 (6)	0.042 (5)	0.11 (1)	−0.009 (4)	0.001 (6)	0.013 (6)
C20	0.050 (6)	0.037 (5)	0.12 (1)	−0.005 (5)	−0.020 (6)	0.011 (6)
O10	0.042 (4)	0.114 (7)	0.036 (4)	−0.003 (4)	0.010 (3)	0.011 (4)
C21	0.039 (6)	0.17 (1)	0.039 (6)	0.003 (7)	0.002 (4)	0.021 (7)
C22	0.052 (7)	0.24 (2)	0.077 (9)	0.03 (1)	0.024 (6)	0.10 (1)
C23	0.049 (6)	0.094 (9)	0.053 (6)	0.008 (6)	0.020 (5)	0.023 (6)
C24	0.038 (5)	0.087 (8)	0.048 (6)	−0.014 (5)	0.009 (4)	−0.008 (5)
O11	0.034 (3)	0.117 (6)	0.038 (3)	−0.006 (4)	−0.001 (3)	−0.036 (4)
C25	0.028 (4)	0.065 (6)	0.058 (6)	−0.010 (4)	0.008 (4)	−0.028 (5)
C26	0.054 (7)	0.16 (2)	0.10 (1)	−0.023 (9)	−0.006 (7)	0.07 (1)
C27	0.061 (7)	0.14 (1)	0.036 (5)	−0.044 (8)	−0.007 (5)	0.018 (6)
C28	0.032 (4)	0.051 (5)	0.034 (4)	−0.008 (4)	0.001 (3)	−0.017 (4)
O12	0.025 (3)	0.049 (4)	0.119 (7)	−0.003 (3)	0.007 (4)	−0.037 (4)
C29	0.021 (4)	0.053 (5)	0.056 (5)	−0.003 (4)	−0.006 (4)	−0.002 (4)
C30	0.026 (4)	0.038 (4)	0.033 (4)	−0.006 (3)	−0.001 (3)	0.003 (3)
C31	0.021 (3)	0.049 (5)	0.039 (4)	0.005 (3)	0.000 (3)	0.002 (4)
C32	0.029 (4)	0.037 (4)	0.035 (4)	0.002 (3)	0.001 (3)	−0.005 (3)

### Geometric parameters (Å, º)

**Table:**

Nb1—Cl1	2.457 (2)	C6B_b—H6D_b	0.9900
Nb1—Cl4	2.457 (2)	C7B_b—H7C_b	0.9900
Nb1—Cl3	2.460 (2)	C7B_b—H7D_b	0.9900
Nb1—Cl2	2.464 (2)	O7—C9	1.423 (9)
Nb1—I1	2.9312 (7)	O7—C12	1.43 (1)
Nb1—Nb4	2.9367 (8)	C9—C10	1.50 (1)
Nb1—Nb5	2.9367 (8)	C9—H9A	0.9900
Nb1—Nb3	2.9394 (8)	C9—H9B	0.9900
Nb1—Nb2	2.9424 (8)	C10—C11	1.50 (1)
Nb2—O1	2.219 (5)	C10—H10A	0.9900
Nb2—Cl9	2.457 (2)	C10—H10B	0.9900
Nb2—Cl1	2.461 (2)	C11—C12	1.49 (1)
Nb2—Cl5	2.461 (2)	C11—H11A	0.9900
Nb2—Cl8	2.473 (2)	C11—H11B	0.9900
Nb2—Nb3	2.8867 (7)	C12—H12A	0.9900
Nb2—Nb5	2.9008 (8)	C12—H12B	0.9900
Nb2—Nb6	2.9381 (8)	O8—C16	1.438 (9)
Nb3—O2	2.233 (4)	O8—C13	1.455 (8)
Nb3—Cl5	2.462 (2)	C13—C14	1.51 (1)
Nb3—Cl10	2.464 (2)	C13—H13A	0.9900
Nb3—Cl2	2.465 (2)	C13—H13B	0.9900
Nb3—Cl6	2.470 (2)	C14—C15	1.51 (1)
Nb3—Nb4	2.9029 (8)	C14—H14A	0.9900
Nb3—Nb6	2.9418 (8)	C14—H14B	0.9900
Nb4—O3	2.232 (5)	C15—C16	1.52 (1)
Nb4—Cl3	2.462 (2)	C15—H15A	0.9900
Nb4—Cl11	2.463 (2)	C15—H15B	0.9900
Nb4—Cl6	2.467 (2)	C16—H16A	0.9900
Nb4—Cl7	2.468 (2)	C16—H16B	0.9900
Nb4—Nb5	2.8923 (8)	O9—C17	1.39 (1)
Nb4—Nb6	2.9366 (8)	O9—C20	1.40 (1)
Nb5—O4	2.209 (5)	C17—C18	1.42 (2)
Nb5—Cl4	2.452 (2)	C17—H17A	0.9900
Nb5—Cl12	2.461 (2)	C17—H17B	0.9900
Nb5—Cl7	2.475 (2)	C18—C19	1.49 (2)
Nb5—Cl8	2.476 (2)	C18—H18A	0.9900
Nb5—Nb6	2.9352 (8)	C18—H18B	0.9900
Nb6—Cl12	2.456 (2)	C19—C20	1.49 (1)
Nb6—Cl9	2.457 (2)	C19—H19A	0.9900
Nb6—Cl11	2.461 (2)	C19—H19B	0.9900
Nb6—Cl10	2.461 (2)	C20—H20A	0.9900
Nb6—I2	2.9563 (7)	C20—H20B	0.9900
O1—H1A	0.8498	O10—C21	1.41 (1)
O1—H1B	0.8500	O10—C24	1.46 (1)
O2—H2A	0.8499	C21—C22	1.43 (2)
O2—H2B	0.8501	C21—H21A	0.9900
O3—H3A	0.8505	C21—H21B	0.9900
O3—H3B	0.8501	C22—C23	1.50 (2)
O4—H4A	0.8500	C22—H22A	0.9900
O4—H4B	0.8498	C22—H22B	0.9900
O5—C1	1.420 (9)	C23—C24	1.46 (1)
O5—C4	1.447 (9)	C23—H23A	0.9900
C1—C2	1.49 (1)	C23—H23B	0.9900
C1—H1C	0.9900	C24—H24A	0.9900
C1—H1D	0.9900	C24—H24B	0.9900
C2—C3	1.51 (1)	O11—C25	1.43 (1)
C2—H2C	0.9900	O11—C28	1.432 (9)
C2—H2D	0.9900	C25—C26	1.46 (2)
C3—C4	1.51 (1)	C25—H25A	0.9900
C3—H3C	0.9900	C25—H25B	0.9900
C3—H3D	0.9900	C26—C27	1.37 (2)
C4—H4C	0.9900	C26—H26A	0.9900
C4—H4D	0.9900	C26—H26B	0.9900
O6A_a—C8A_a	1.48 (3)	C27—C28	1.50 (1)
O6A_a—C5A_a	1.53 (6)	C27—H27A	0.9900
C5A_a—C6A_a	1.55 (7)	C27—H27B	0.9900
C5A_a—H5A_a	0.9900	C28—H28A	0.9900
C5A_a—H5B_a	0.9900	C28—H28B	0.9900
C6A_a—C7A_a	1.46 (8)	O12—C32	1.409 (9)
C6A_a—H6A_a	0.9900	O12—C29	1.44 (1)
C6A_a—H6B_a	0.9900	C29—C30	1.51 (1)
C7A_a—C8A_a	1.5 (1)	C29—H29A	0.9900
C7A_a—H7A_a	0.9900	C29—H29B	0.9900
C7A_a—H7B_a	0.9900	C30—C31	1.50 (1)
C8A_a—H8A_a	0.9900	C30—H30A	0.9900
C8A_a—H8B_a	0.9900	C30—H30B	0.9900
O6B_b—C5B_b	1.2 (1)	C31—C32	1.51 (1)
C5B_b—C6B_b	1.6 (1)	C31—H31A	0.9900
C5B_b—H5C_b	0.9900	C31—H31B	0.9900
C5B_b—H5D_b	0.9900	C32—H32A	0.9900
C6B_b—C7B_b	1.6 (1)	C32—H32B	0.9900
C6B_b—H6C_b	0.9900		
			
Cl1—Nb1—Cl4	88.15 (6)	Nb2—O1—H1A	126.4
Cl1—Nb1—Cl3	164.99 (6)	Nb2—O1—H1B	135.5
Cl4—Nb1—Cl3	89.96 (6)	H1A—O1—H1B	91.8
Cl1—Nb1—Cl2	88.73 (6)	Nb3—O2—H2A	109.8
Cl4—Nb1—Cl2	165.04 (6)	Nb3—O2—H2B	127.2
Cl3—Nb1—Cl2	89.28 (6)	H2A—O2—H2B	103.6
Cl1—Nb1—I1	82.67 (4)	Nb4—O3—H3A	109.8
Cl4—Nb1—I1	81.57 (4)	Nb4—O3—H3B	126.8
Cl3—Nb1—I1	82.32 (4)	H3A—O3—H3B	108.6
Cl2—Nb1—I1	83.52 (4)	Nb5—O4—H4A	109.4
Cl1—Nb1—Nb4	141.61 (5)	Nb5—O4—H4B	109.3
Cl4—Nb1—Nb4	96.11 (4)	H4A—O4—H4B	109.5
Cl3—Nb1—Nb4	53.40 (4)	C1—O5—C4	109.5 (6)
Cl2—Nb1—Nb4	95.42 (4)	O5—C1—C2	107.2 (7)
I1—Nb1—Nb4	135.71 (2)	O5—C1—H1C	110.3
Cl1—Nb1—Nb5	95.59 (4)	C2—C1—H1C	110.3
Cl4—Nb1—Nb5	53.17 (4)	O5—C1—H1D	110.3
Cl3—Nb1—Nb5	95.17 (4)	C2—C1—H1D	110.3
Cl2—Nb1—Nb5	141.75 (4)	H1C—C1—H1D	108.5
I1—Nb1—Nb5	134.73 (2)	C1—C2—C3	103.0 (6)
Nb4—Nb1—Nb5	59.00 (2)	C1—C2—H2C	111.2
Cl1—Nb1—Nb3	94.90 (4)	C3—C2—H2C	111.2
Cl4—Nb1—Nb3	141.48 (4)	C1—C2—H2D	111.2
Cl3—Nb1—Nb3	95.84 (4)	C3—C2—H2D	111.2
Cl2—Nb1—Nb3	53.40 (4)	H2C—C2—H2D	109.1
I1—Nb1—Nb3	136.92 (2)	C4—C3—C2	103.2 (6)
Nb4—Nb1—Nb3	59.21 (2)	C4—C3—H3C	111.1
Nb5—Nb1—Nb3	88.35 (2)	C2—C3—H3C	111.1
Cl1—Nb1—Nb2	53.30 (4)	C4—C3—H3D	111.1
Cl4—Nb1—Nb2	94.48 (4)	C2—C3—H3D	111.1
Cl3—Nb1—Nb2	141.71 (4)	H3C—C3—H3D	109.1
Cl2—Nb1—Nb2	95.36 (4)	O5—C4—C3	106.2 (6)
I1—Nb1—Nb2	135.96 (2)	O5—C4—H4C	110.5
Nb4—Nb1—Nb2	88.31 (2)	C3—C4—H4C	110.5
Nb5—Nb1—Nb2	59.13 (2)	O5—C4—H4D	110.5
Nb3—Nb1—Nb2	58.78 (2)	C3—C4—H4D	110.5
O1—Nb2—Cl9	80.9 (1)	H4C—C4—H4D	108.7
O1—Nb2—Cl1	81.0 (1)	C8A_a—O6A_a—C5A_a	107 (3)
Cl9—Nb2—Cl1	161.91 (6)	O6A_a—C5A_a—C6A_a	99 (3)
O1—Nb2—Cl5	79.4 (1)	O6A_a—C5A_a—H5A_a	112.0
Cl9—Nb2—Cl5	89.21 (6)	C6A_a—C5A_a—H5A_a	112.0
Cl1—Nb2—Cl5	88.78 (6)	O6A_a—C5A_a—H5B_a	112.0
O1—Nb2—Cl8	82.3 (1)	C6A_a—C5A_a—H5B_a	112.0
Cl9—Nb2—Cl8	86.65 (6)	H5A_a—C5A_a—H5B_a	109.7
Cl1—Nb2—Cl8	89.63 (6)	C7A_a—C6A_a—C5A_a	105 (4)
Cl5—Nb2—Cl8	161.62 (6)	C7A_a—C6A_a—H6A_a	110.7
O1—Nb2—Nb3	133.5 (1)	C5A_a—C6A_a—H6A_a	110.7
Cl9—Nb2—Nb3	97.19 (4)	C7A_a—C6A_a—H6B_a	110.7
Cl1—Nb2—Nb3	96.17 (4)	C5A_a—C6A_a—H6B_a	110.7
Cl5—Nb2—Nb3	54.11 (4)	H6A_a—C6A_a—H6B_a	108.8
Cl8—Nb2—Nb3	144.22 (4)	C6A_a—C7A_a—C8A_a	102 (5)
O1—Nb2—Nb5	136.4 (1)	C6A_a—C7A_a—H7A_a	111.4
Cl9—Nb2—Nb5	95.69 (4)	C8A_a—C7A_a—H7A_a	111.4
Cl1—Nb2—Nb5	96.44 (4)	C6A_a—C7A_a—H7B_a	111.4
Cl5—Nb2—Nb5	144.18 (4)	C8A_a—C7A_a—H7B_a	111.4
Cl8—Nb2—Nb5	54.17 (4)	H7A_a—C7A_a—H7B_a	109.3
Nb3—Nb2—Nb5	90.07 (2)	O6A_a—C8A_a—C7A_a	108 (3)
O1—Nb2—Nb6	134.2 (1)	O6A_a—C8A_a—H8A_a	110.2
Cl9—Nb2—Nb6	53.29 (4)	C7A_a—C8A_a—H8A_a	110.2
Cl1—Nb2—Nb6	144.78 (4)	O6A_a—C8A_a—H8B_a	110.2
Cl5—Nb2—Nb6	96.26 (4)	C7A_a—C8A_a—H8B_a	110.2
Cl8—Nb2—Nb6	95.58 (4)	H8A_a—C8A_a—H8B_a	108.5
Nb3—Nb2—Nb6	60.66 (2)	O6B_b—C5B_b—C6B_b	109 (8)
Nb5—Nb2—Nb6	60.35 (2)	O6B_b—C5B_b—H5C_b	109.9
O1—Nb2—Nb1	134.2 (1)	C6B_b—C5B_b—H5C_b	109.9
Cl9—Nb2—Nb1	144.85 (4)	O6B_b—C5B_b—H5D_b	109.9
Cl1—Nb2—Nb1	53.20 (4)	C6B_b—C5B_b—H5D_b	109.9
Cl5—Nb2—Nb1	96.78 (4)	H5C_b—C5B_b—H5D_b	108.3
Cl8—Nb2—Nb1	96.90 (5)	C7B_b—C6B_b—C5B_b	91 (7)
Nb3—Nb2—Nb1	60.56 (2)	C7B_b—C6B_b—H6C_b	113.5
Nb5—Nb2—Nb1	60.34 (2)	C5B_b—C6B_b—H6C_b	113.5
Nb6—Nb2—Nb1	91.58 (2)	C7B_b—C6B_b—H6D_b	113.5
O2—Nb3—Cl5	80.2 (1)	C5B_b—C6B_b—H6D_b	113.5
O2—Nb3—Cl10	81.6 (1)	H6C_b—C6B_b—H6D_b	110.8
Cl5—Nb3—Cl10	87.84 (6)	C6B_b—C7B_b—H7C_b	111.0
O2—Nb3—Cl2	80.1 (1)	C6B_b—C7B_b—H7D_b	111.0
Cl5—Nb3—Cl2	89.33 (6)	H7C_b—C7B_b—H7D_b	109.0
Cl10—Nb3—Cl2	161.77 (5)	C9—O7—C12	108.6 (6)
O2—Nb3—Cl6	81.7 (1)	O7—C9—C10	106.2 (7)
Cl5—Nb3—Cl6	161.93 (6)	O7—C9—H9A	110.5
Cl10—Nb3—Cl6	89.20 (5)	C10—C9—H9A	110.5
Cl2—Nb3—Cl6	87.92 (6)	O7—C9—H9B	110.5
O2—Nb3—Nb2	134.3 (1)	C10—C9—H9B	110.5
Cl5—Nb3—Nb2	54.08 (4)	H9A—C9—H9B	108.7
Cl10—Nb3—Nb2	96.13 (4)	C11—C10—C9	104.8 (7)
Cl2—Nb3—Nb2	96.76 (4)	C11—C10—H10A	110.8
Cl6—Nb3—Nb2	143.99 (4)	C9—C10—H10A	110.8
O2—Nb3—Nb4	135.6 (1)	C11—C10—H10B	110.8
Cl5—Nb3—Nb4	144.11 (4)	C9—C10—H10B	110.8
Cl10—Nb3—Nb4	96.56 (4)	H10A—C10—H10B	108.9
Cl2—Nb3—Nb4	96.26 (4)	C12—C11—C10	103.5 (8)
Cl6—Nb3—Nb4	53.95 (4)	C12—C11—H11A	111.1
Nb2—Nb3—Nb4	90.04 (2)	C10—C11—H11A	111.1
O2—Nb3—Nb1	133.5 (1)	C12—C11—H11B	111.1
Cl5—Nb3—Nb1	96.83 (4)	C10—C11—H11B	111.1
Cl10—Nb3—Nb1	144.85 (4)	H11A—C11—H11B	109.0
Cl2—Nb3—Nb1	53.39 (4)	O7—C12—C11	103.1 (7)
Cl6—Nb3—Nb1	95.83 (4)	O7—C12—H12A	111.2
Nb2—Nb3—Nb1	60.66 (2)	C11—C12—H12A	111.2
Nb4—Nb3—Nb1	60.35 (2)	O7—C12—H12B	111.2
O2—Nb3—Nb6	134.9 (1)	C11—C12—H12B	111.2
Cl5—Nb3—Nb6	96.15 (4)	H12A—C12—H12B	109.1
Cl10—Nb3—Nb6	53.28 (4)	C16—O8—C13	109.3 (6)
Cl2—Nb3—Nb6	144.95 (4)	O8—C13—C14	105.6 (6)
Cl6—Nb3—Nb6	96.33 (4)	O8—C13—H13A	110.6
Nb2—Nb3—Nb6	60.53 (2)	C14—C13—H13A	110.6
Nb4—Nb3—Nb6	60.32 (2)	O8—C13—H13B	110.6
Nb1—Nb3—Nb6	91.56 (2)	C14—C13—H13B	110.6
O3—Nb4—Cl3	81.6 (1)	H13A—C13—H13B	108.7
O3—Nb4—Cl11	80.0 (1)	C13—C14—C15	102.3 (7)
Cl3—Nb4—Cl11	161.58 (6)	C13—C14—H14A	111.3
O3—Nb4—Cl6	81.8 (1)	C15—C14—H14A	111.3
Cl3—Nb4—Cl6	88.54 (6)	C13—C14—H14B	111.3
Cl11—Nb4—Cl6	88.29 (6)	C15—C14—H14B	111.3
O3—Nb4—Cl7	79.9 (1)	H14A—C14—H14B	109.2
Cl3—Nb4—Cl7	88.21 (6)	C14—C15—C16	102.1 (7)
Cl11—Nb4—Cl7	89.13 (6)	C14—C15—H15A	111.3
Cl6—Nb4—Cl7	161.75 (6)	C16—C15—H15A	111.3
O3—Nb4—Nb5	134.2 (1)	C14—C15—H15B	111.3
Cl3—Nb4—Nb5	96.25 (4)	C16—C15—H15B	111.3
Cl11—Nb4—Nb5	96.97 (4)	H15A—C15—H15B	109.2
Cl6—Nb4—Nb5	143.93 (4)	O8—C16—C15	105.7 (6)
Cl7—Nb4—Nb5	54.31 (4)	O8—C16—H16A	110.6
O3—Nb4—Nb3	135.8 (1)	C15—C16—H16A	110.6
Cl3—Nb4—Nb3	96.72 (4)	O8—C16—H16B	110.6
Cl11—Nb4—Nb3	96.07 (4)	C15—C16—H16B	110.6
Cl6—Nb4—Nb3	54.02 (4)	H16A—C16—H16B	108.7
Cl7—Nb4—Nb3	144.23 (4)	C17—O9—C20	109.7 (8)
Nb5—Nb4—Nb3	89.92 (2)	O9—C17—C18	110 (1)
O3—Nb4—Nb6	133.4 (1)	O9—C17—H17A	109.6
Cl3—Nb4—Nb6	145.05 (4)	C18—C17—H17A	109.6
Cl11—Nb4—Nb6	53.36 (4)	O9—C17—H17B	109.6
Cl6—Nb4—Nb6	96.51 (4)	C18—C17—H17B	109.6
Cl7—Nb4—Nb6	96.41 (4)	H17A—C17—H17B	108.1
Nb5—Nb4—Nb6	60.47 (2)	C17—C18—C19	106.1 (9)
Nb3—Nb4—Nb6	60.50 (2)	C17—C18—H18A	110.5
O3—Nb4—Nb1	134.9 (1)	C19—C18—H18A	110.5
Cl3—Nb4—Nb1	53.33 (4)	C17—C18—H18B	110.5
Cl11—Nb4—Nb1	145.08 (4)	C19—C18—H18B	110.5
Cl6—Nb4—Nb1	95.94 (4)	H18A—C18—H18B	108.7
Cl7—Nb4—Nb1	96.49 (4)	C20—C19—C18	104.3 (9)
Nb5—Nb4—Nb1	60.50 (2)	C20—C19—H19A	110.9
Nb3—Nb4—Nb1	60.44 (2)	C18—C19—H19A	110.9
Nb6—Nb4—Nb1	91.72 (2)	C20—C19—H19B	110.9
O4—Nb5—Cl4	80.6 (1)	C18—C19—H19B	110.9
O4—Nb5—Cl12	81.0 (1)	H19A—C19—H19B	108.9
Cl4—Nb5—Cl12	161.67 (6)	O9—C20—C19	106.4 (9)
O4—Nb5—Cl7	81.0 (1)	O9—C20—H20A	110.5
Cl4—Nb5—Cl7	89.82 (6)	C19—C20—H20A	110.5
Cl12—Nb5—Cl7	87.28 (6)	O9—C20—H20B	110.5
O4—Nb5—Cl8	80.9 (1)	C19—C20—H20B	110.5
Cl4—Nb5—Cl8	88.27 (6)	H20A—C20—H20B	108.7
Cl12—Nb5—Cl8	88.88 (6)	C21—O10—C24	107.8 (8)
Cl7—Nb5—Cl8	161.88 (6)	O10—C21—C22	108.4 (9)
O4—Nb5—Nb4	135.1 (1)	O10—C21—H21A	110.0
Cl4—Nb5—Nb4	97.38 (4)	C22—C21—H21A	110.0
Cl12—Nb5—Nb4	95.53 (4)	O10—C21—H21B	110.0
Cl7—Nb5—Nb4	54.06 (4)	C22—C21—H21B	110.0
Cl8—Nb5—Nb4	144.02 (4)	H21A—C21—H21B	108.4
O4—Nb5—Nb2	134.9 (1)	C21—C22—C23	103 (1)
Cl4—Nb5—Nb2	95.66 (4)	C21—C22—H22A	111.1
Cl12—Nb5—Nb2	97.30 (4)	C23—C22—H22A	111.1
Cl7—Nb5—Nb2	144.04 (4)	C21—C22—H22B	111.1
Cl8—Nb5—Nb2	54.06 (4)	C23—C22—H22B	111.1
Nb4—Nb5—Nb2	89.97 (2)	H22A—C22—H22B	109.1
O4—Nb5—Nb6	134.3 (1)	C24—C23—C22	102.7 (9)
Cl4—Nb5—Nb6	145.07 (4)	C24—C23—H23A	111.2
Cl12—Nb5—Nb6	53.26 (4)	C22—C23—H23A	111.2
Cl7—Nb5—Nb6	96.28 (4)	C24—C23—H23B	111.2
Cl8—Nb5—Nb6	95.58 (4)	C22—C23—H23B	111.2
Nb4—Nb5—Nb6	60.51 (2)	H23A—C23—H23B	109.1
Nb2—Nb5—Nb6	60.45 (2)	O10—C24—C23	104.3 (8)
O4—Nb5—Nb1	134.0 (1)	O10—C24—H24A	110.9
Cl4—Nb5—Nb1	53.35 (4)	C23—C24—H24A	110.9
Cl12—Nb5—Nb1	144.97 (4)	O10—C24—H24B	110.9
Cl7—Nb5—Nb1	96.31 (4)	C23—C24—H24B	110.9
Cl8—Nb5—Nb1	96.97 (4)	H24A—C24—H24B	108.9
Nb4—Nb5—Nb1	60.50 (2)	C25—O11—C28	109.1 (7)
Nb2—Nb5—Nb1	60.53 (2)	O11—C25—C26	106.8 (8)
Nb6—Nb5—Nb1	91.75 (2)	O11—C25—H25A	110.4
Cl12—Nb6—Cl9	89.46 (6)	C26—C25—H25A	110.4
Cl12—Nb6—Cl11	88.43 (6)	O11—C25—H25B	110.4
Cl9—Nb6—Cl11	164.93 (6)	C26—C25—H25B	110.4
Cl12—Nb6—Cl10	164.84 (6)	H25A—C25—H25B	108.6
Cl9—Nb6—Cl10	89.32 (5)	C27—C26—C25	110 (1)
Cl11—Nb6—Cl10	88.82 (5)	C27—C26—H26A	109.8
Cl12—Nb6—Nb5	53.44 (4)	C25—C26—H26A	109.8
Cl9—Nb6—Nb5	94.80 (4)	C27—C26—H26B	109.8
Cl11—Nb6—Nb5	95.91 (4)	C25—C26—H26B	109.8
Cl10—Nb6—Nb5	141.71 (4)	H26A—C26—H26B	108.2
Cl12—Nb6—Nb4	94.55 (4)	C26—C27—C28	107.9 (9)
Cl9—Nb6—Nb4	141.64 (4)	C26—C27—H27A	110.1
Cl11—Nb6—Nb4	53.42 (4)	C28—C27—H27A	110.1
Cl10—Nb6—Nb4	95.77 (4)	C26—C27—H27B	110.1
Nb5—Nb6—Nb4	59.02 (2)	C28—C27—H27B	110.1
Cl12—Nb6—Nb2	96.47 (4)	H27A—C27—H27B	108.4
Cl9—Nb6—Nb2	53.27 (4)	O11—C28—C27	105.9 (7)
Cl11—Nb6—Nb2	141.81 (4)	O11—C28—H28A	110.6
Cl10—Nb6—Nb2	94.90 (4)	C27—C28—H28A	110.6
Nb5—Nb6—Nb2	59.19 (2)	O11—C28—H28B	110.6
Nb4—Nb6—Nb2	88.39 (2)	C27—C28—H28B	110.6
Cl12—Nb6—Nb3	141.76 (4)	H28A—C28—H28B	108.7
Cl9—Nb6—Nb3	95.75 (4)	C32—O12—C29	110.4 (6)
Cl11—Nb6—Nb3	95.13 (4)	O12—C29—C30	103.7 (7)
Cl10—Nb6—Nb3	53.38 (4)	O12—C29—H29A	111.0
Nb5—Nb6—Nb3	88.34 (2)	C30—C29—H29A	111.0
Nb4—Nb6—Nb3	59.19 (2)	O12—C29—H29B	111.0
Nb2—Nb6—Nb3	58.81 (2)	C30—C29—H29B	111.0
Cl12—Nb6—I2	81.77 (4)	H29A—C29—H29B	109.0
Cl9—Nb6—I2	82.38 (4)	C31—C30—C29	101.6 (6)
Cl11—Nb6—I2	82.55 (4)	C31—C30—H30A	111.5
Cl10—Nb6—I2	83.09 (4)	C29—C30—H30A	111.5
Nb5—Nb6—I2	135.19 (2)	C31—C30—H30B	111.5
Nb4—Nb6—I2	135.95 (2)	C29—C30—H30B	111.5
Nb2—Nb6—I2	135.65 (2)	H30A—C30—H30B	109.3
Nb3—Nb6—I2	136.47 (2)	C30—C31—C32	104.3 (6)
Nb1—Cl1—Nb2	73.50 (5)	C30—C31—H31A	110.9
Nb1—Cl2—Nb3	73.21 (4)	C32—C31—H31A	110.9
Nb1—Cl3—Nb4	73.26 (5)	C30—C31—H31B	110.9
Nb5—Cl4—Nb1	73.48 (5)	C32—C31—H31B	110.9
Nb2—Cl5—Nb3	71.81 (4)	H31A—C31—H31B	108.9
Nb4—Cl6—Nb3	72.03 (4)	O12—C32—C31	106.1 (6)
Nb4—Cl7—Nb5	71.63 (4)	O12—C32—H32A	110.5
Nb2—Cl8—Nb5	71.77 (4)	C31—C32—H32A	110.5
Nb2—Cl9—Nb6	73.44 (5)	O12—C32—H32B	110.5
Nb6—Cl10—Nb3	73.34 (4)	C31—C32—H32B	110.5
Nb6—Cl11—Nb4	73.21 (4)	H32A—C32—H32B	108.7
Nb6—Cl12—Nb5	73.30 (4)		
			
C4—O5—C1—C2	14.9 (9)	C20—O9—C17—C18	10 (2)
O5—C1—C2—C3	−29.6 (9)	O9—C17—C18—C19	3 (2)
C1—C2—C3—C4	32.4 (8)	C17—C18—C19—C20	−13 (2)
C1—O5—C4—C3	6.3 (9)	C17—O9—C20—C19	−18 (2)
C2—C3—C4—O5	−24.3 (8)	C18—C19—C20—O9	18 (2)
C8A_a—O6A_a—C5A_a—C6A_a	−30 (3)	C24—O10—C21—C22	3 (2)
O6A_a—C5A_a—C6A_a—C7A_a	45 (5)	O10—C21—C22—C23	−24 (2)
C5A_a—C6A_a—C7A_a—C8A_a	−41 (5)	C21—C22—C23—C24	36 (2)
C5A_a—O6A_a—C8A_a—C7A_a	7 (4)	C21—O10—C24—C23	20 (1)
C6A_a—C7A_a—C8A_a—O6A_a	21 (5)	C22—C23—C24—O10	−34 (1)
O6B_b—C5B_b—C6B_b—C7B_b	−32 (10)	C28—O11—C25—C26	6 (1)
C12—O7—C9—C10	20 (1)	O11—C25—C26—C27	−9 (2)
O7—C9—C10—C11	4 (1)	C25—C26—C27—C28	8 (2)
C9—C10—C11—C12	−25 (1)	C25—O11—C28—C27	−1 (1)
C9—O7—C12—C11	−35 (1)	C26—C27—C28—O11	−5 (2)
C10—C11—C12—O7	36 (1)	C32—O12—C29—C30	27 (1)
C16—O8—C13—C14	12.1 (8)	O12—C29—C30—C31	−36.7 (8)
O8—C13—C14—C15	−31.5 (8)	C29—C30—C31—C32	33.1 (8)
C13—C14—C15—C16	38.1 (8)	C29—O12—C32—C31	−6 (1)
C13—O8—C16—C15	12.5 (9)	C30—C31—C32—O12	−17.8 (9)
C14—C15—C16—O8	−31.7 (9)		

### Hydrogen-bond geometry (Å, º)

**Table:**

*D*—H···*A*	*D*—H	H···*A*	*D*···*A*	*D*—H···*A*
O1—H1*A*···O5	0.85	1.79	2.642 (7)	180
O1—H1*B*···O6*A*_*a*	0.85	1.75	2.60 (3)	178
O1—H1*B*···O6*B*_*b*	0.85	1.83	2.68 (5)	173
O2—H2*A*···O7	0.85	1.83	2.639 (7)	158
O2—H2*B*···O8	0.85	1.78	2.634 (7)	180
O3—H3*B*···O9	0.85	1.75	2.601 (8)	179
O3—H3*A*···O10	0.85	1.88	2.637 (8)	148
O4—H4*B*···O11	0.85	1.92	2.613 (8)	138
O4—H4*A*···O12	0.85	2.09	2.530 (8)	112
C3—H3*C*···Cl4^i^	0.99	2.94	3.930 (8)	177
C3—H3*D*···Cl12^ii^	0.99	2.95	3.657 (8)	130
C9—H9*B*···Cl11^iii^	0.99	2.98	3.642 (8)	125
C14—H14*A*···Cl6^iv^	0.99	2.97	3.931 (9)	165
C23—H23*A*···Cl5^v^	0.99	2.99	3.74 (1)	134
C31—H31*B*···Cl8^vi^	0.99	2.79	3.778 (8)	176

Symmetry codes: (i) −*x*+3/2, *y*+1/2, *z*; (ii) *x*+1/2, *y*, −*z*+1/2; (iii) *x*+1/2, −*y*+1/2, −*z*+1; (iv) −*x*+1, −*y*+1, −*z*+1; (v) *x*−1/2, −*y*+1/2, −*z*+1; (vi) *x*−1/2, *y*, −*z*+1/2.
